# From rapalogs to anti-aging formula

**DOI:** 10.18632/oncotarget.18033

**Published:** 2017-05-22

**Authors:** Mikhail V. Blagosklonny

**Affiliations:** ^1^ Cell Stress Biology, Roswell Park Cancer Institute, Buffalo, NY, USA

**Keywords:** lifespan, longevity, rejuvenation, health, diseases

## Abstract

Inhibitors of mTOR, including clinically available rapalogs such as rapamycin (Sirolimus) and Everolimus, are gerosuppressants, which suppress cellular senescence. Rapamycin slows aging and extends life span in a variety of species from worm to mammals. Rapalogs can prevent age-related diseases, including cancer, atherosclerosis, obesity, neurodegeneration and retinopathy and potentially rejuvenate stem cells, immunity and metabolism. Here, I further suggest how rapamycin can be combined with metformin, inhibitors of angiotensin II signaling (Losartan, Lisinopril), statins (simvastatin, atorvastatin), propranolol, aspirin and a PDE5 inhibitor. Rational combinations of these drugs with physical exercise and an anti-aging diet (Koschei formula) can maximize their anti-aging effects and decrease side effects.

At first, the discovery of anti-aging properties of rapamycin was met with skepticism because it challenged the dogma that aging is a decline driven by molecular damage caused by free radicals. By now, rapamycin has been proven to be an anti-aging drug. In contrast, anti-oxidants failed in clinical trials [1–9] and the dogma was shattered [1, 2, 10–18]. In the last decade, anti-aging effects of rapamycin have been confirmed. Anti-aging doses and schedules can be extrapolated from animal studies. Well-tolerated doses with minimal side effects can be deducted based on clinical use of rapalogs. So optimal anti-aging doses/schedules can be suggested. Given that rapamycin consistently extends maximal lifespan in mice, rapamycin will likely allow mankind to beat the current record of human longevity, which is 122 years. Yet, rapamycin will not extend life span as much as we might wish to.

Now is the time for anti-aging drug combinations. For example, metformin is currently undergoing re-purposing as an anti-aging agent. Several other existing drugs can be re-purposed. Now we can design an anti-aging formula, using drugs available for human use. However, we must first discuss the link between growth, aging and age-related diseases.

## MTOR: from growth to aging

It was theoretically predicted that stimulation of mitogenic/growth pathways in arrested or quiescent cells must lead to senescence [19]. This conversion from quiescence to senescence is called geroconversion [20–22]. Cellular senescence is a futile growth, a continuation of cellular growth when actual growth is restricted [21, 23, 24]. Growth-stimulation of arrested cells causes their hypertrophy and hyperfunctions (for example, hyper-secretory phenotype or SASP in senescent fibroblasts).

This can be applied to organismal aging. When developmental growth is completed, then mTOR (mammalian Target of Rapamycin) and some other signaling pathways) drives organismal aging [1, 15, 25, 26]. These pathways stimulate cellular functions, leading to hyperfunctions (for example, hypertension). Secondary, hyperfunctions can lead to loss of functions [1, 27]. Hyperfunction theory links growth, aging and age-related diseases [1]. Suppression of aging prevents or delays age-related diseases [17, 28–30].

## Age-related diseases are manifestations of advanced aging

Age-related pathologies and conditions include atherosclerosis, hypertension, osteoporosis, obesity, insulin-resistance and type II diabetes, cancer, macular degeneration, Parkinson and Alzheimer's diseases as well as menopause in women, and many changes in the appearance that are not called diseases (baldness, for example) and presbyopia (a condition that resembles nearsightedness). Stroke, myocardial infarction, heart fibrillation, broken hip, renal and other organs failure are consequences of age-related pathology [17, 28, 31].

In brief, age-related diseases are both manifestations of advanced aging and causes of death. Aging is the sum of age-related diseases, syndromes and symptoms ranging from wrinkles and presbyopia to stroke and cancer metastasis. Of course, age-related diseases can occur in young patients with either genetic predisposition or due to environmental hazards. However, each of these diseases will develop in the aging organism, even without any predispositions and hazards, if the organism would live long enough. Since aging is not programmed, these diseases develop at different speeds. For example, menopause (in women) and presbyopia develop fast and strike all aging humans. Whereas, Alzheimer disease develops slowly and an elderly person can die from cancer or stroke before Alzheimer disease takes place [17, 28].

**Figure 1:**
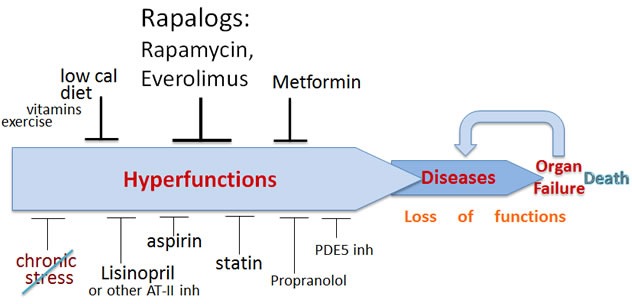
Schema of aging and its pharmacological suppression Aging is an increase in the probability of death. Aging is a continuation of developmental growth, when the development is stopped but signaling pathways (such as mTOR) remain active. Chronic cellular overactivation increases cellular functions (secretion, synthesis, metabolism, contraction, aggregation, lipid accumulation and so on), leading to systemic hyperfuntions such as hypertension and other diseases of aging. Hyperfunction, manifested as age-related diseases, causes organ damage and loss of functions. Aging consists from subclinical hyperfunction, diseases and loss of function/organ failure. Anti-aging drugs inhibit signaling pathways, decreasing hyperfunction, slowing down aging and delaying diseases and death. The most important drugs are shown in larger fonts.

In brief, animals die from age-related diseases, which are manifestations of advanced aging (Figure 1). If a drug delays ALL age-related diseases, it is a classic anti-aging drug because it will extend life span by delaying causes of death.

## What are (and are not) anti-aging drugs?

Both insulin and metformin are used to treat type II diabetes. Insulin can save a patient in a diabetic coma. Metformin would not perform such a miracle. However, insulin cannot prevent diabetes, whereas metformin can. Insulin can also accelerate some diseases, whereas metformin decelerates them. (See metformin section for references). Insulin can foster cancer and obesity. Metformin prevents cancer and decreases obesity. Insulin activates the mTOR pathway, a key pathway of aging. Metformin indirectly inhibits the mTOR pathway. Insulin is a pro-aging drug, whereas metformin is an anti-aging drug.

## Criteria for potential anti-aging drugs

1. A drug that prolongs life span in model organisms preferably in mammals.
2. A drug that prevents or delays several age-related diseases in mammals.
3. A drug that suppresses cellular geroconversion from quiescence to senescence

These criteria overlap each other. If an intervention extends life span, it must delay age-related diseases. Animals die from age-related diseases. For example, calorie restriction (CR) delays all diseases of aging and extends life span. One may say that CR extends life span by delaying diseases. One may say that CR delays diseases by slowing down aging. Both interpretations are correct. By the way, CR deactivates the nutrient-sensing pathway, known as TOR (Target of Rapamycin) [32]. According to all 3 criteria, rapamycin and other rapalogs are ultimate anti-aging drugs.

## Rapalogs: Rapamycin (Sirolimus/Rapamune) and Everalimus

Rapalogs include rapamycin, known in the clinic as Sirolimus or Rapamune, everolimus, temsirolimus (a rapamycin prodrug) and deforolimus (Ridaforolimus). Rapalogs are prescription drugs taken by organ transplant recipients and cancer patients.

1. Rapamycin prolongs life span in mice [33–45] at doses that have no noticeable side effects [46–59].
2. Rapalogs prevent age-related diseases in mice as well as in other mammals including non-human primates and humans. As examples: rapamycin prevents atherosclerosis [60–64], neurodegeneration and retinopathy [65–67] and cardiopathy [68] in rodents. Rapalogs prevent cancer in mice and humans [34, 37, 38, 40, 41, 69–86]. Rapamycin decreases obesity in mice and humans [87–89]. As predicted [1], rapalogs rejuvenate immunity, improve immune response in aging mice [90] and humans [30, 91, 92]. Prevention of all age-related diseases by rapalogs was discussed in detail [1, 2, 13, 14, 17, 18, 24, 26, 29, 32 93–95],
3. Rapalogs suppress cellular geroconversion from quiescence to senescence [20–23, 90, 96–110].
4. Rapamycin suppresses aging, age-related pathologies in model organisms:the yeast [111, 112], the fly [113–118] and the worm [119].

According to all criteria, rapalogs are anti-aging drugs. Importantly, rapalogs have minimal side effects, which can be reversed [120–122]. In some studies, rapamycin improves metabolic parameters [46, 49, 52, 56, 57, 88, 123]. Rapalogs have been used in healthy volunteers [124, 125] and even in pregnant women without detrimental effects [126, 127]. Recently, rapamycin was investigated as an anti-aging drug in humans [128].

In transplant patients, rapalogs prevent cancer [69–78]. This is a very good “side effect”. In certain strains of mice, rapamycin causes symptoms of “hunger” pseudo-diabetes described 150 years ago by Claude Bernard [129]. “Hunger pseudo-diabetes” is not a disease. It is a beneficial condition during full fasting [130, 31]. During starvation glucose must be spared for the brain, so the body becomes insulin-resistant and insulin production decreases. These metabolic changes are benevolent and therefore fasting is good for the health. In fact, rapamycin prevents complications of diabetes (nephropathy), while increasing glucose levels in genetically diabetic mice [131]. Prevention of diabetic complications with rapamycin has been discussed [31]. Unfortunately, basic scientists misinterpreted starvation-like effects of rapamycin as type 2 diabetes. Based on this misinterpretation, some gerontologists believe that this precludes the use of rapalogs in humans. Fortunately, rapalogs are already widely used in humans. In transplant patients, there is a very slight increase of diabetes manifestations in some studies but not in other studies [132, 133]. Noteworthy, diabetes is common in transplant patients regardless of rapalog treatment, so this group of patients is not representative. What about healthy individuals? Acute administration of rapamycin reverses food-induced insulin resistance [134]. In other words, rapamycin increases insulin sensitivity in healthy people.

Of course, it is possible that chronic administration of rapamycin may cause beta-cell disfunction and diabetes in some genetically-predisposed humans, like it was observed in some mice strains [135]. This does not preclude the use of rapamycin as an anti-aging. Simply, glucose levels should be monitored and rapamycin can be discontinued, if glucose levels increase. Unlike transplant patients, healthy individuals can discontinue rapamycin at any time. And finally, to “mitigate” these worries, rapamycin can be combined with metformin.

## Metformin

Metformin is one of the most commonly used drugs to treat type II diabetes. Before metformin, its analog – phenformin – was used for type II diabetes. Phenformin was removed from the market because of the fear of its rare side effect: lactic acidosis. The incidence of lactic acidosis for metformin is lower than for phenformin.

Since 1972, Russian scientist, Vladimir Dilman, and his co-workers Anisimov, Bernstein and others, demonstrated that phenformin and metformin slow down aging, decrease obesity, prevent cancer and extend lifespan in rodents [136–146]. Furthermore, phenformin and metformin were administered to cancer patients [147]. For many years, these publications were ignored because it was believed that aging is driven by molecular damage and cannot be suppressed by anti-diabetic drugs. The anti-aging effect of metformin and phenformin was explained by the hyper-function theory of aging [1].

In fact, metformin increases lifespan in mice [143] and C. elegans [148, 149]. Metformin prevents cancer and some other age-related diseases in humans. [150–161]. Furthermore, metformin decreases all-cause mortality in diabetic patients [156] and reduces the risk of cognitive decline and dementia [157]. Also, metformin is used to increase fertility [162,163].

Thus, metformin (a) extends life span in worms and rodents and decreases all-cause mortality in humans (b) prevents several age-related diseases in rodents and humans. Yet, metformin did not increase lifespan in some model organisms. It does not increase lifespan in mice in some studies. The spectrum of metformin-responsive diseases is narrower than those for rapamycin. As a monotherapy, the life-extending effect of metformin may be modest, but it can be combined with rapamycin and other drugs.

Two agents may even cancel each other's potential side effects. For example, whereas metformin can increase lactate production, rapamycin decreases it [164]. This is important because phenformin was removed from the market because of the fear of lactate acidosis, caused by lactate production. On the other hand, metformin is expected to reduce manifestation of benevolent glucose-intolerance, if rapamycin will cause these manifestations, in a minority of individuals. A combination of rapamycin and metformin is also studied for cancer therapy [165, 166].

## Inhibitors of angiotensin II

Angiotensin II receptor blockers (ARB) such as Valsartan, Telmisartan, Losartan and angiotensin-converting enzyme (ACE) inhibitors such as Captopril, Lisinopril, Enalapril, Ramipril are widely used to treat hypertension. Hypertension is a clear-cut disease of hyperfunction. Angiotensin II, a hormone, is involved in age-related diseases in mammals [167, 168]. Disruption of the angiotensin II receptor increases longevity in mice [169]. Variations of the angiotensin II receptor gene are associated with longevity in humans [170]. Inhibitors of angiotensin II double lifespan of hypertensive rats [171, 172]. This dramatic (100%) increase is in part due to the anti-hypertensive effect. Yet, in healthy (those with normal blood pressure) rats, long-term treatment with enalapril decreases weight and prolongs life span dramatically [173]. In humans, inhibitors of angiotensin II prevent cardiac hypertrophy and organ fibrosis [168], [174], a hallmark of aging. In some studies, long-term use of ARBs was associated with a lower incidence of cancer [175]. Enalapril and perindopril did not decrease blood pressure in patients with normal blood pressure [176]. Importantly, angiotensin-converting enzyme inhibitors or angiotensin receptor blockers are beneficial in normotensive atherosclerotic patients [177].

## Aspirin

Aspirin or acetylsalicylic acid, an inhibitor of cyclooxygenase (COX), is one of the most widely used non-prescription drugs. Aspirin inhibits inflammation. Pro-inflammation (an example of hyperfunction) is a hallmark of aging [178, 179]. Aspirin also inhibits hyper-functional platelets, preventing thrombosis and atherosclerosis. Inhibition of hyper-active platelets prevents cardiovascular diseases and cancer [180].

Aspirin prolongs life span in Drosophila [181], C elegans [182, 183] and mice [184]. Aspirin reverses glucose intolerance in rats [185]. Anti-aging activities of aspirin have been discussed [105, 186, 187].

Aspirin is used to prevent age-related diseases including cardiovascular diseases and cancer in humans. Numerous studies have demonstrated benefits, although doses and duration of treatment remain uncertain. For example, 600 mg aspirin per day for 25 months decreased the incidence of cancer in carriers of hereditary colorectal cancer [188]. In another study, 300 mg a day for 5 years prevented colorectal cancer [189]. Long-term daily use of aspirin decreases the incidence of colorectal, prostate, and breast cancers [190]. In some studies, regular, long-term aspirin use reduced the risk of colorectal cancer [191], whereas, in other studies, occasional use of aspirin prevented vascular diseases and cancer [192]. In one of the most comprehensive studies, aspirin at doses between 75 and 325 mg/day for 5 years significantly decreased cancer incidence [192]. It slightly increased chances of gastric bleeding [192, 193]. It was estimated that, by preventing cancer, aspirin can save more lives than lost lives due to potential side effects [192, 193].

## Statins

Statins, such as atorvastatin (Lipitor), simvastatin and lovastatin, decrease blood cholesterol levels and thus decelerate atherosclerosis, preventing cardiovascular diseases [194]. Statins are beneficial in hypertension [195–197]. Statins can decrease the incidence of some cancers [198]. Simvastatin increases mean and maximum lifespan of Drosophila [199]. Statins increase life span in progeroid mice [200]. Yet, in another study, a statin did not prolong life span in mice [36]. Statins prolong lifespan by 2 years in humans treated at ages 78 to 85 [194]. Among the very old, the extension of life was independent of cholesterol levels [201].

Noteworthy, statins can prevent rapamycin-induced dyslipidemia [202]. This benevolent dyslipidemia is caused by lipolysis and inhibition of lipoproteins uptake by the tissues (see Figure 2 in [94]). Dyslipidemia is reversible by itself [64]. Yet, it is easier to combine rapamycin and statins than to prove that dyslipidemia is totally harmless. Statins can “mitigate the fear” of this rapamycin-induced “side effect”. Statins have side-effects, which are, in rare cases, dangerous. Statins, which are prescription drugs, are available in grocery stores without prescription as natural products. Lovastatin is a natural compound found in oyster mushrooms and red yeast rice, a food supplement. Red yeast rice is often combined with berberine and policosanol, natural food supplements.

## Beta-blockers

Beta-blockers are widely used to treat hypertension and heart diseases. Propranolol, a non-selective beta-adrenergic blocker, prevents cancer [203–206] and hepatic steatosis [207]. Propranolol is used out-of-label to decrease anxiety. Metoprolol and nebivolol increase the mean and median life span of male mice, by 10% and 6.4% and extend Drosophila life span [208].

## PDE5 inhibitors

Phosphodiesterase 5 (PDE5) degrades cGMP. PDE5 inhibitors, including sildenafil (Viagra), tadalafil (Cialis), vardenafil (Levitra), and avanafil (Stendra), are widely known for treatment of erectile dysfunction (ED). While sexual stimulation causes cGMP synthesis, PDE5 inhibitors cause its accumulation, relaxing corpus cavernosum and penile arteries. In addition, cGMP relaxes the tone of prostate muscle cells and decreases prostate inflammation. Tadalafil is effective and well-tolerated therapy for benign prostate hyperplasia (BPH) [209]. PDE5 inhibitors, such as long-acting tadalafil, can be added to anti-aging drug mixture because ED and BPH are most prevalent age-related conditions in aging men. Also, there is a fundamental reason to consider PDE5 inhibitors as anti-aging medicines (in both men and women). The key word is “relax”. Senescent cells are tense and stressed. Cellular senescence is manifested by hyper-function. On the organism levels hyper-function is translated in hypertension, hyperplasia, hyperlipidemia and so on. By ‘relaxing the tension’, PDE5 inhibitors may slow senescent-associated pathology. PFE5 inhibitors cause anti-vasoconstriction, anti-proliferative and anti-inflammatory effects. Not co-incidentally, PDE5 inhibitors are already approved or investigated for therapy of diverse age-related diseases and conditions. Sildenafil and tadalafil are approved for pulmonary arterial hypertension [210]. PDE5 inhibitors are under investigation for heart hypertrophy, myocardial infarction, cancer, neurodegenerative diseases, cystic fibrosis, diabetes, obesity and metabolic syndrome [210–213]. Inhibition of PDE5 increases levels of cGMP and hydrogen sulfate. These signaling molecules increase life span in C elegans [214, 215].

PDE5 inhibitors are remarkably safe for everyday use for a long term. In most studies, 5 mg tadalafil once a day had minimal side effect and improved BPH and ED [216]. Furthermore, even 40 mg of tadalafil is used in the treatment of pulmonary arterial hypertension without serious side effects [217]. PDE5 inhibition improves beta-cell function in metabolic syndrome [218, 219]. This may mitigate potential side effect of rapamycin on beta-cells.

## Doxocycline

Doxocycline, broad-spectrum antibiotics of the tetracycline class, extends life span in C elegans [220] and Drosophila [221, 222]. Doxycycline suppresses tumor growth and metastasis in mice [223, 224]. Importantly, doxycycline is a component of an anti-metastatic combination, which includes doxycyclin, aspirin, lisin and mifepristone [225].

## Melatonin

Melatonin, a hormone, which is sold as a non-prescription sleeping pill, increases life span and decreases cancer incidence in animals in some studies [226–229]. Yet, other studies were inconclusive.

## Experimental gerosuppressants

Like rapalogs, pan-mTOR inhibitors suppress geroconversion [107–109, 110, 230]. They suppress geroconversion at concentrations lower than anti-cancer doses [230]. Low doses of pan-mTOR inhibitors have not been yet tested in mice to determine the effect of life span. Since these drugs are not yet approved for human use, we will not discuss them here. MDM-2 inhibitors [96, 231] MEK inhibitors [232] and S6K inhibitors [233] also suppress geroconversion in cell type-specific manner. Gerosuppression and tumor-suppression are two sides of one coin, so not surprisingly, they were intended as anticancer drugs [234–236]. At anti-cancer doses, gerosuppressants inhibit cell proliferation. Therefore, anti-aging doses should be lower than standard anti-cancer doses. Alternatively, gerosuppressants should be used intermittently: high therapeutic doses followed by treatment-free periods.

## Polypill

Polypill is a fixed-dose combination of antiplatelet (aspirin), anti-hypertensive drugs (lisinopril and beta-blocker), and a statin [237–239]. Polypill also may include additional anti-hypertensive drugs [240] as well as folic acid [237]. Polypill showed life-extending activity in high-risk elderly individuals [241, 242]. It was calculated that polypill may reduce strokes and ischemic heart disease by over 80% in individuals at risk for cardiovascular diseases [242]. Polypill may prevent cardiovascular disease and strokes [243, 244]. Polypill includes 4 ‘anti-aging’ drugs (statin, aspirin, beta-blocker, angiotensin II inhibitor such as lisinopril). Yet, this combination was created to prevent cardiovascular diseases, not to slow aging. Therefore, Polypill does not include two main anti-aging components: a rapalog and metformin. Nevertheless, Polypill is used in ‘healthy’ aging humans in order to extend life span by preventing diseases. It needs to be combined with rapamycin and metformin, to maximize lifespan extension.

## Anti-aging combinations

Rapamycin (or another rapalog) should be a cornerstone of anti-aging combinations (Figure 1), given its universal anti-aging effect and the ability to delay almost all diseases of aging.

Rapamycin and metformin: Both drugs extend lifespan in animals and have non- overlapping effects. In addition, they may, in theory, cancel possible metabolic side-effects of each other. As we discussed here (see rapamycin section) as well as in [31, 130], rapamycin in different settings may either increase or decrease insulin sensitivity. Similarly, calorie restriction increases insulin sensitivity, whereas severe calorie restriction (starvation) decreases it [129, 245]. In any case, rapamycin prolongs life span, indicating that insulin resistance is benevolent [130]. Unfortunately, the fear of this benevolent ‘side effect’ is delaying applications of rapamycin for prevention of age-related diseases. The simplest approach is to monitor glucose levels in individuals taking rapamycin. In addition, metformin, an anti-diabetic drug that reverses insulin resistance, could be combined with rapamycin.

And vice verse, metformin may potentially increase blood lactate levels. Rapamycin decreases lactate production [164]. Each drug prolongs lifespan in mice, prevents cancer, atherosclerosis, and other diseases of aging.

Rapamycin and statins: Rapamycin promotes lipolysis increasing blood levels of fatty acids. This, in turn, increases levels of lipoproteins produced by the liver. Rapamycin-induced hyperlipidemia is benevolent and reversible. Still, statins are already used to prevent rapamycin-induced hyperlipidemia [202, 246, 247]

Rapamycin and physical exercise: Similarly, physical exercise may be useful to prevent rapamycin-induced hyperlipidemia because fatty acids are used by the muscle during physical exercise.

### Rapamycin and low-calorie diet or intermittent fasting

- During fasting, the organism depends on lipolysis and ketogenesis. Rapamycin stimulates these processes. Fatty acids and ketone bodies will be used by the muscle and the brain, respectively.
- Fasting decreases glucose, potentially mitigating possible rapamycin-induced hyperglycemia

The effects of rapamycin and calorie-restriction are not identical and may be additive [248, 249]. Calorie restriction and intermittent fasting extend life span. A low-calorie diet can be supplemented with vitamins (poly-vitamins plus, B3, B12 and D3), minerals and even essential amino and fatty acids, if needed, to avoid malnutrition.

It is commonly suggested that certain food is beneficial because it is rich in some ‘useful’ ingredients: anti-oxidants, vitamins, minerals, essential fatty acids. Yet, food is also rich in calories. Using supplements, there is no need to eat food because it is “rich in something” (vitamin C, D or promega-3). Eating food for any essential component will bring calories along with the essential component. Food rich in vitamins could be substituted with vitamins alone.

## Rapamycin and PDE5 inhibitors

Cialis is approved for treatment of BPH and rapamycin treat BPH in preclinical studies. Rapamycin can decrease beta-cell function, whereas PDE5 inhibitors can increase it.

## Rapamycin-based mixtures

- Rapamycin plus metformin (especially in insulin-resistant and obese people, metformin is indicated).
- Rapamycin plus Lisinopril (or other angiotensin II-inhibitor) plus propranolol. Like in Polypill, these prescription drugs may be used at ½ doses in normotensive individuals. Hypertensive patients may require full doses.
- Rapamycin plus Statin (such as lovastatin, simvastatin and atorvastatin)
- Rapamycin plus Statin plus metformin. This combination with rapamycin may be the most attractive for people with metabolic alterations: hyperlipidemia, obesity, insulin resistance.
- Rapamycin plus polypill-like combination (Lisinopril, propranolol, aspirin, statin). This is especially attractive in people with atherosclerosis given that rapamycin prevents atherosclerosis too.
- Rapamycin plus Lisinopril (or other Angiotensin II-inhibitor) + propranolol + aspirin + statin + metformin + PDE5inhibitor. This is a comprehensive 7-drug combination.

## Doses and schedules

In the 7-drug anti-aging combination, rapamycin, metformin, lisinopril (or its equivalent), a statin, a PDE5 inhibitor and propranolol are prescription drugs (in the USA). So I will not discuss doses and schedules here. They should be determined for each individual *individually*. Polypill composition provides the hint on doses of 4 drugs in healthy individuals. The doses of rapamycin are beyond the scope of this article. Mixtures of anti-aging drugs should be further complemented with physical exercise and low-calorie diet or intermittent diet. Additional drugs such as melatonin may be considered. The 7-drug combination can be tested in mice, especially in mice on high fat diet and in cancer-prone mice. If started late in life, the experiments will take just several months to evaluate the effect on lifespan and cancer incidence as well as weight, blood pressure, glucose, insulin, triglycerides and leptin. In humans, the treatment program can be initiated regardless of any pre-clinical studies, because all 7 drugs are approved for human use and some of them such as aspirin and statin are widely used for disease prevention anyway. The only what is needed is to watch for side effects. Especially, heart rate, blood pressure and glucose levels should be monitored.

## From past to the future

As stated in 2006, “… rapamycin, is already approved for clinical use, available and can be used immediately … to slow down senescence and to prevent diseases.”[1]. It was suggested that in intermittent schedules, rapamycin will be effective, yet lack side effects. Pulse-treatment was suggested to improve wound healing and rejuvenate stem cells and immunity [1, 27]. After 10 years, this suggestion remains unchanged. The implementation of anti-aging drugs to live longer and to delay age-related diseases was discussed in detail [94, 250, 251].

Now, the time is for the anti-aging formula, which combines around 7 drugs with diet and physical exercise. The anti-aging formula is ready for human use. If one will wait until the life-extending effect will be shown in others, this individual will not be alive by the time of the result. Human clinical trials are needed to optimize the doses and schedules. However, unless we participate in clinical trials ourselves, we will not know how long participants will live because they are expected to outlive non-participants. If we want to live longer we should be participants in clinical trials. In the best scenario, this might allow us to live long enough to benefit from future discoveries of anti-aging remedies. Experimental anti-aging drugs such as pan-mTOR inhibitors might be approved for future anti-aging formulas. Finally, if mTOR-driven aging will be abolished, anti-oxidants may become useful to treat post-aging syndrome [245]. And step-by-step, humanity will extend life span.
